# The neutrophil-to-lymphocyte ratio could be a good diagnostic marker and predictor of relapse in patients with adult-onset Still's disease: A STROBE-compliant retrospective observational analysis.

**DOI:** 10.1097/MD.0000000000007546

**Published:** 2017-07-21

**Authors:** Ji-Yeoun Seo, Chang-Hee Suh, Ju-Yang Jung, Ar-Reum Kim, Ji Won Yang, Hyoun-Ah Kim

**Affiliations:** Department of Rheumatology, Ajou University School of Medicine, Worldcup-ro, Yeongtong-gu, Suwon, Korea.

**Keywords:** adult-onset Still's disease, diagnosis, macrophage activation syndrome, neutrophil-to-lymphocyte ratio

## Abstract

The neutrophil-to-lymphocyte ratio (NLR) is the proportion of absolute neutrophil count to lymphocytes on routine complete blood count (CBC) tests, and has been studied as a simple marker of the systemic inflammatory response. This study was performed to investigate whether the NLR could be used as a tool to diagnose and predict prognosis in cases of adult-onset Still's disease (AOSD).

We retrospectively reviewed 164 patients with suspected AOSD. Among 164 patients with suspected AOSD, 37 patients received another diagnosis (such as viral infection) and were compared with the 127 patients who received a diagnosis of AOSD. Laboratory tests including CBCs, ferritin, erythrocyte sedimentation rate (ESR), C-reactive protein (CRP) level, and the NLR were evaluated.

AOSD patients showed higher neutrophil counts, lower lymphocyte counts, higher NLRs, and higher levels of ferritin, ESR, and CRP than non-AOSD patients (all *P* < .001). In receiver operating characteristic (ROC) curve analysis of the NLR for diagnosis of AOSD, the area under the curve (AUC) was highest at 0.967 (95% CI = 0.940–0.993) with a cutoff value of 3.08. The cutoff value showed the greatest sensitivity (91.7%), specificity (68.4%), and AUC value (0.967) as a diagnostic tool for AOSD. The NLR and treatment appeared to be significant prognostic factors for relapse, but only age showed a significant relationship with death. Furthermore, the NLR was significantly higher in patients with macrophage activation syndrome than in hemophagocytic lymphohistiocytosis (HLH) patients (*P* = .007). In ROC analysis, the NLR with a cutoff value of 5.86 showed a sensitivity of 89.4%, specificity of 87.8%, and AUC of 0.794.

The NLR can be used as a diagnostic tool and predictor of relapse in AOSD, and for differential diagnosis of HLH.

## 1 Introduction

Adult-onset Still's disease (AOSD), a rare systemic inflammatory disease, is an adult form of systemic juvenile idiopathic arthritis (JIA). AOSD shows nonspecific manifestations with high fever, arthralgia, arthritis, salmon-colored skin rash, lymphadenopathy, hepatosplenomegaly, and serositis. The laboratory findings of acute AOSD are leukocytosis, thrombocytosis, and elevated acute-phase reactants and liver enzymes. Early diagnosis of AOSD is difficult because there are no clear-cut diagnostic symptoms or characteristic serological biomarkers. Diagnosis of AOSD has been based on the Yamaguchi or Fautrel criteria only after excluding infections, malignancies, especially malignant lymphoma, and other rheumatic diseases.^[1–3]^

Systemic inflammation is associated with neutrophilia, thrombocytosis, lymphopenia, and normochromic anemia. Therefore, the features of circulating blood cell components have been suggested to be biomarkers for assessment of inflammatory activity.^[4]^ The neutrophil-to-lymphocyte ratio (NLR) is the proportion of absolute neutrophil count to lymphocytes on routine complete blood count (CBC) tests. There have been a number of studies regarding the NLR as a simple marker of the systemic inflammatory response.^[4,5]^ The NLR is associated with coronary heart disease, poor prognosis in patients with pancreatitis, malignancy, and chronic inflammatory diseases, such as ulcerative colitis and rheumatoid arthritis (RA).^[5–10]^ AOSD has laboratory abnormalities common to systemic inflammation, such as neutrophilic leukocytosis and lymphopenia. However, there have been no studies regarding the relationship between AOSD and the NLR as a biomarker for prognosis or disease activity.

In this study, we retrospectively reviewed clinical symptoms, laboratory findings, and clinical courses using the NLR in AOSD patients. We investigated whether the NLR could be used to diagnose and predict prognosis in cases of AOSD.

## 2 Methods

### 2.1 Subjects

We found 185 patients with our hospital computer system with code of adult-onset Still's disease, and 21 patients were excluded from the study because of insufficient data or missing data. We retrospectively reviewed 164 patients with suspected AOSD at the time of the initial visit to Ajou University Hospital between 1999 and 2017. Patients meeting 2 or more of the major Yamaguchi criteria were selected, after exclusion of hematological, infectious, and other autoimmune diseases.^[1]^ During the evaluation for diagnosis, 37 patients received another diagnosis, such as viral infection, palindromic rheumatism, lupus-like disease, or Kikuchi's disease. Finally, 127 patients who received a diagnosis of AOSD were compared with the 37 patients who did not receive a diagnosis of AOSD.

Of the 127 AOSD patients, 12 (9.4%) were diagnosed with macrophage activation syndrome (MAS). MAS is characterized by high fever, generalized lymphadenopathy, hemorrhagic symptoms, hepatosplenomegaly, and central nervous system dysfunction. Pancytopenia, and increased levels of liver enzymes, ferritin, LDH, triglycerides, D-dimer, and soluble interleukin-2 (IL-2), is seen in MAS.^[11]^ MAS in all patients was confirmed by bone marrow, lymph node, or liver biopsy. We found 19 hemophagocytic lymphohistiocytosis (HLH) patients without autoimmune disease during the same period and compared them with the AOSD patients with MAS. This study was approved by the Institutional Review Board of our hospital (AJIRB-MED-MED-17–065).

### 2.2 Variables

All clinical data were based on electronic medical records. Clinical characteristics, including age, gender, clinical symptoms and signs, follow-up periods, treatments, and outcomes, were evaluated. Laboratory tests, including CBCs, ferritin, liver function tests, erythrocyte sedimentation rate (ESR), C-reactive protein (CRP), lactate dehydrogenase (LDH), and the NLR, were evaluated. We also obtained systemic disease scores using the method described by Pouchot et al^[12]^ on a scale ranging from 0 to 12, with 1 point awarded for each of the following symptoms: fever, typical rash, sore throat, lymphadenopathy, leukocytosis ≥ 15,000/mm^2^, myalgia, abdominal pain, hepatomegaly, splenomegaly, pneumonia, pleuritis, and pericarditis.

### 2.3 Statistical analysis

All data are expressed as means ± standard deviation, and *P* < .05 was considered statistically significant. Clinical characteristics were compared between the AOSD group and non-AOSD group, AOSD with MAS group, and HLH group using Pearson's χ^2^ test for categorical variables and independent *t*-test for continuous variables. Receiver operating characteristic (ROC) analysis was used for evaluation of diagnostic markers in AOSD. The optimal cutoff values of several markers including NLR that the best distinguished AOSD group from the non-AOSD group was determined with the maximum value of Youden's index, which was calculated by sensitivity + 1-specificity.^[13,14]^ The overall diagnostic accuracy and predictive ability were estimated based on the area under the curve (AUC) which is reported with its standard error. A multivariable analysis was performed with significant markers from ROC curves to determine which of them are independently associated with the diagnosis for AOSD. Pearson's correlation was applied to assess correlations between disease activity markers and the NLR in AOSD patients. We used the independent *t*-test to compare the NLR according to clinical symptoms in AOSD patients. Statistical analyses were performed using SPSS software (ver. 23.0; SPSS Inc., Chicago, IL).

## 3 Results

### 3.1 Clinical characteristics of AOSD patients

A total of 164 patients with suspected AOSD at the initial visit to the hospital were evaluated. During the evaluation for diagnosis, 37 patients received another diagnosis. Twelve patients were diagnosed to palindromic rheumatism, 11 patients to lupus-like disease, 4 to viral exanthema, 2 to systemic lupus erythematosus (SLE), 2 to drug eruption, 2 to Kikuch's disease, 1 to thyroid cancer, 1 to breast cancer, 1 to hypereosinophilic syndrome, and 1 to human immunodeficiency virus infection. The laboratory results indicated that all the patients were in the initial stage of active disease prior to corticosteroid treatment. Table 1 shows the clinical characteristics of AOSD patients and non-AOSD patients (a group initially suspected of having, but then shown not to have, AOSD). The median duration of follow up was 13 months in AOSD patients. The main clinical symptoms in AOSD included fever (98.4%), arthralgia (85.8%), skin rash (74.8%), and sore throat (52.8%). The frequencies of clinical features were not significantly different between AOSD and non-AOSD patients, except fever and skin rash, which were observed more frequently in AOSD patients. The mean NLR in the AOSD group was 15.94 ± 16.64. AOSD patients showed higher neutrophil counts, lower lymphocyte counts, and higher NLRs than non-AOSD patients (all *P* < .001). Higher levels of ferritin, ESR, and CRP were observed in AOSD patients (all *P* < .001). There were no significant differences in any other characteristic between the 2 groups.

**Table 1:**
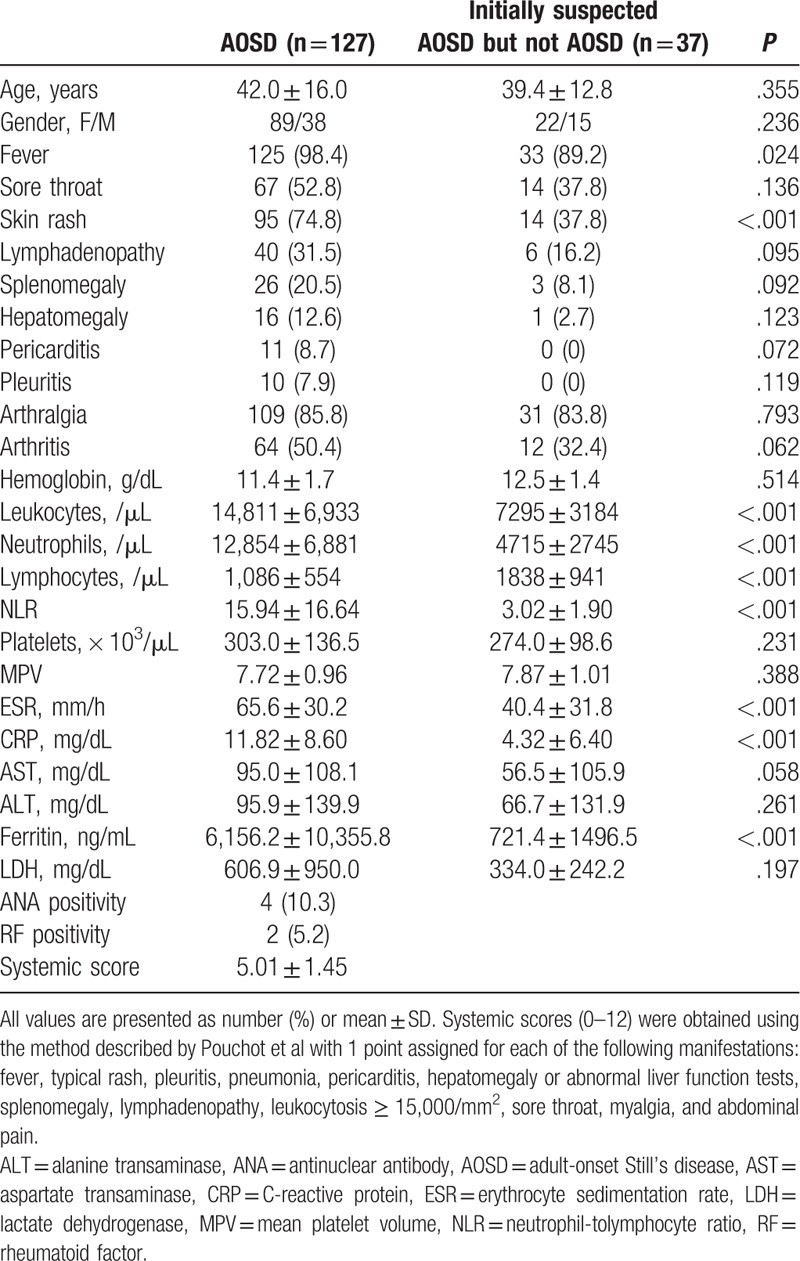
Clinical characteristics of patients.

The AOSD patients were treated with corticosteroids (95.3%), nonsteroidal anti-inflammatory drugs (NSAIDs) (82.7%), hydroxychloroquine (69.3%), and methotrexate (52%). Some AOSD patients were injected with intravenous immunoglobulin (IVIG) (19.7%) and tumor necrosis factor (TNF) inhibitors (6.3%) with systemic corticosteroids. During the follow-up period, 4 patients (3.1%) in the AOSD group died and 35 (27.6%) relapsed (Table 2). One patient expired due to fulminant hepatitis related AOSD, and 3 patients expired due to pneumonia with sepsis during the follow-up. Their median duration of follow up was 36 months, and their mean age was 57.0 ± 18.8.

**Table 2:**
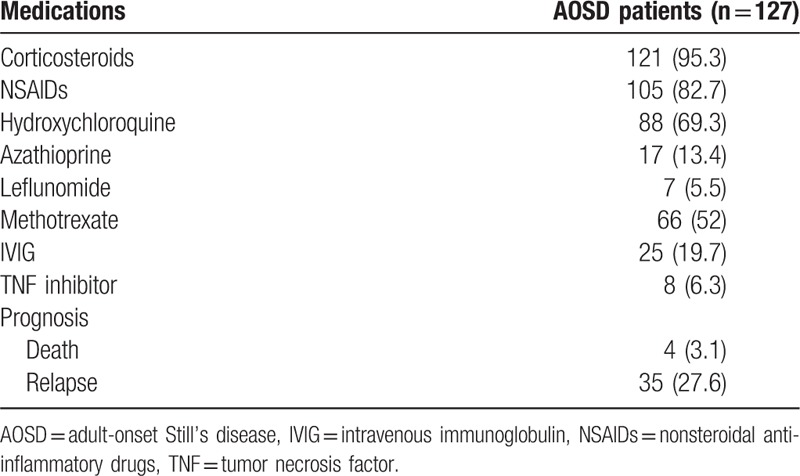
Treatment and prognosis of AOSD patients during follow-up.

Of the AOSD patients, 66 (52.0%) patients had AOSD of the monophasic pattern, 34 (26.8%) disease of the polycyclic pattern, 23 (18.1%) disease of the chronic articular pattern, and 4 (3.1%) patients who expired. When we compared NLR levels by AOSD clinical course (monocyclic vs polycyclic and chronic articular), those levels did not differ between 2 groups (15.3 ± 18.2 vs 17.0 ± 15.4, *P* = .573).

Multivariate logistic regression was performed to determine prognostic factors for relapse in AOSD. Patients were treated according to 3 steps: patients used only corticosteroids or NSAIDs in step 1, with disease modifying antirheumatic drugs (DMARDs) used—regardless of the use of corticosteroids—in step 2; patients were treated with corticosteroids, as well as TNF inhibitors or IVIG, regardless of the use of DMARDs in step 3. The NLR and treatment appeared to be significant prognostic factors for relapse (Table 3). The patients treated with step 1 were relapsed more than the patients treated with step 2 or 3.

**Table 3:**
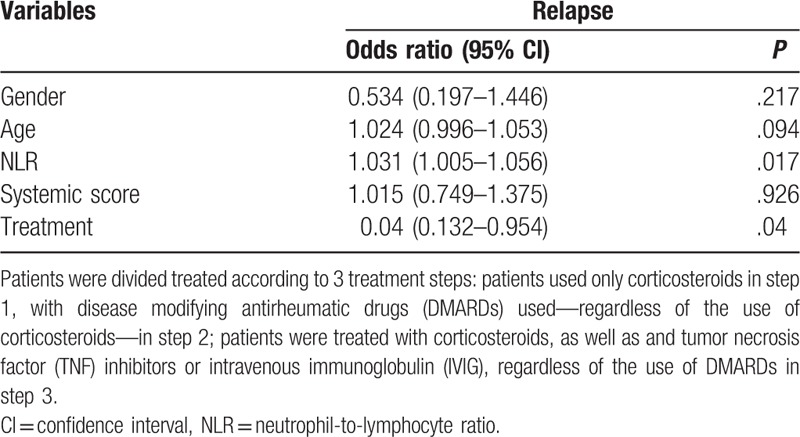
Variables independently associated with relapse in patients with adult-onset Still's disease.

### 3.2 The NLR as a diagnostic tool and a marker for evaluation of disease activity in AOSD patients

**Figure 1:**
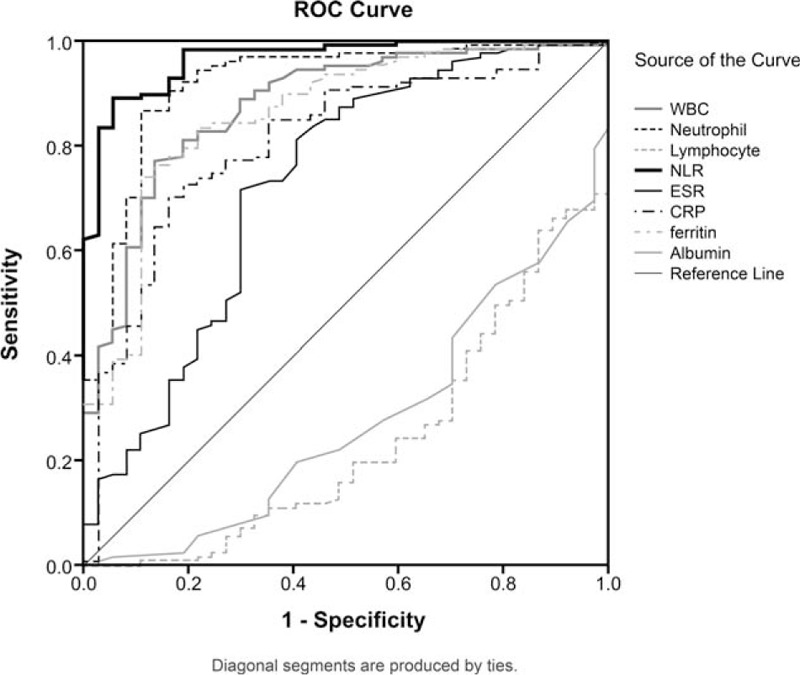
Receiver operating characteristic (ROC) curves for leukocytes, neutrophils, lymphocytes, the neutrophil-to-lymphocyte ratio (NLR), erythrocyte sedimentation rate (ESR), and C-reactive protein (CRP), ferritin, and albumin levels in adult-onset Still's disease (AOSD) patients and non-AOSD patients (a group initially suspected of having, but then shown not to have, AOSD). The ROC curve values were 0.876 for leukocytes (95% CI = 0.813–0.940), 0.920 for neutrophils (95% CI = 0.866–0.973), 0.237 for lymphocytes (95% CI = 0.152–0.322), 0.967 for the NLR (95% CI = 0.940–0.993), 0.726 for ESR (95% CI = 0.625–0.828), 0.808 for CRP (95% CI = 0.729–0.887), 0.860 for ferritin (95% CI = 0.791–0.930), and 0.278 for albumin (95% CI = 0.189–0.366). AOSD = adult-onset Still's disease, CI = confidence interval, CRP = C-reactive protein, ESR = erythrocyte sedimentation rate, NLR = neutrophil-to-lymphocyte ratio, ROC = receiver operating characteristic.

We used ROC curves with several inflammatory markers to evaluate diagnostic tools for AOSD. In ROC analysis of leukocytes, neutrophils, lymphocytes, the NLR, ESR, CRP, ferritin, and albumin, the areas under the curve (AUC) were 0.876 (95% CI = 0.813–0.940), 0.920 (95% CI = 0.866–0.973), 0.237 (95% CI = 0.152–0.322), 0.967 (95% CI = 0.940–0.993), 0.726 (95% CI = 0.625–0.828), 0.808 (95% CI = 0.729–0.887), 0.860 (95% CI = 0.791–0.930), and 0.278 (95% CI = 0.189–0.366), respectively (Fig. 1). We performed a multivariable analysis with significant markers from ROC curves to determine which of them are independently associated with the diagnosis for AOSD (Table 4). The NLR was significantly associated for diagnosis of AOSD (odds ratio = 2.336, *P* < .001). The optimal cutoff value that best distinguished AOSD from non-AOSD was determined at the maximum value, which was estimated by sensitivity + 1-specificity in the ROC curves. Among the variables, the NLR, with a cutoff value of 3.08, showed the greatest sensitivity (91.7%), specificity (68.4%), and AUC value (0.967) as a diagnostic tool for AOSD.

**Table 4:**
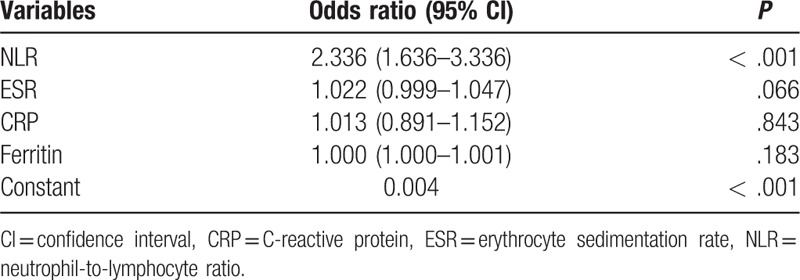
Variables distinguished AOSD from non-AOSD with multivariable analysis.

Among 95 patients with skin rash in AOSD, 17 patients (17.9%) had atypical skin manifestatsions, such as persistent pruritic papules and plaques. We compared the NLR levels between the patients with typical skin manifestations (14.7 ± 13.0) and the patients with atypical skin mainfestations (13.2 ± 10.9). However, there was no difference of the NLR levels between them.

We also evaluated the NLR as a marker of disease activity, using Pearson's correlation and several disease activity markers of AOSD. In AOSD patients, the NLR was weakly correlated with the systemic score (*r* = 0.258, *P* = .004), and CRP (*r* = 0.245, *P* = .005), and ferritin levels (*r* = 0.291, *P* = .001) (Table 5). Therefore, there were no strong correlations between the NLR and disease activity markers. We also compared the NLR according to disease manifestations, but there were no significant differences according to the presence or absence of each symptom (data not shown).

**Table 5:**
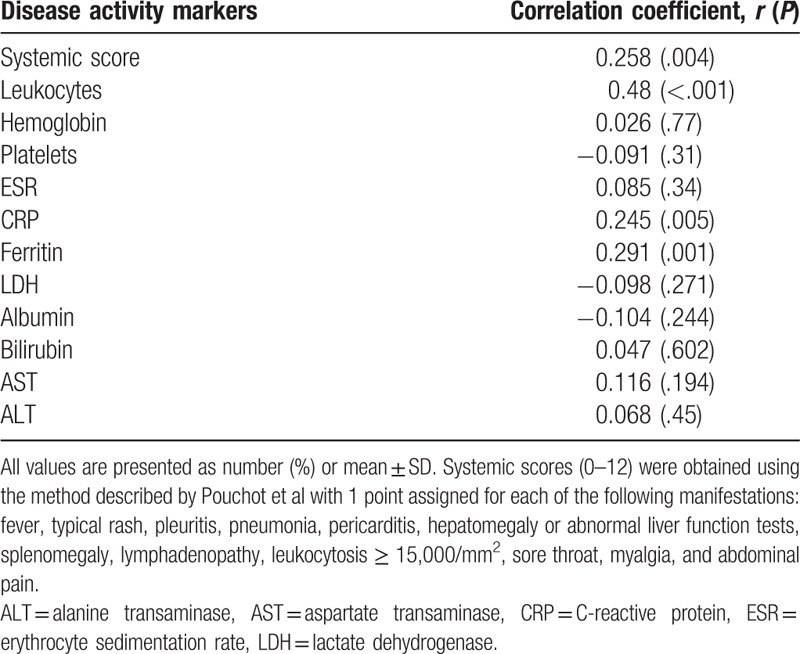
Correlation between disease activity markers and the neutrophil-to-lymphocyte ratio in adult-onset Still's disease.

### 3.3 Comparison of AOSD patients with MAS and HLH patients

**Figure 2:**
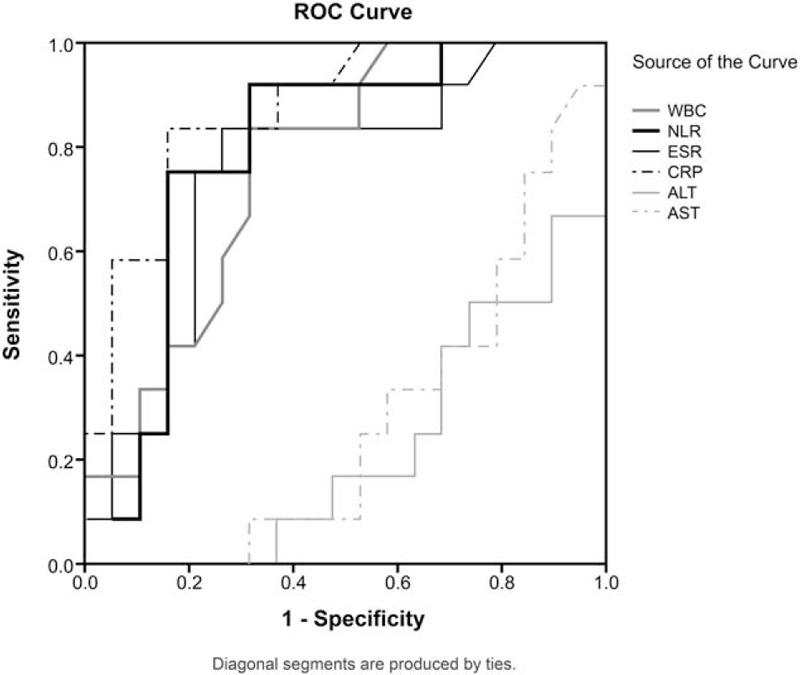
The ROC curves of leukocytes, the NLR, ESR, and CRP, aspartate transaminase (AST), and alanine transaminase (ALT) levels in AOSD patients with macrophage activation syndrome and hemophagocytic lymphohistiocytosis (HLH) patients. The ROC curve values were 0.761 for leukocytes (95% CI = 0.594–0.928), 0.794 for the NLR (95% CI = 0.628–0.959), 0.757 for ESR (95% CI = 0.577–0.936), 0.871 for CRP (95% CI = 0.745–0.996), 0.274 for AST (95% CI = 0.096–0.452), and 0.219 for ALT (95% CI = 0.057–0.382). ALT = alanine transaminase, AST = aspartate transaminase, AOSD = adult-onset Still's disease, CI = confidence interval, CRP = C-reactive protein, ESR = erythrocyte sedimentation rate, HLH = hemophagocytic lymphohistiocytosis, NLR = neutrophil-to-lymphocyte ratio, ROC = receiver operating characteristic.

Twelve MAS patients were compared with nineteen HLH patients during the same period (Table 6). Lymphadenopathy (41.7% vs 100%, respectively, *P* < .001) and hepatomegaly (41.7% vs 73.7%, respectively, *P* = .008) were significantly more frequent in HLH patients. The incidence of arthralgia was significantly higher in AOSD patients with MAS than in HLH patients (91.7% vs 52.6%, respectively, *P* = .024) (Table 6). With regard to laboratory data, patients with MAS showed significantly higher leukocyte, neutrophil, ESR, CRP, AST, and ALT levels. Furthermore, the NLR was significantly higher in patients with MAS than in HLH patients (4.84 vs 3.19, respectively, *P* = .006). In ROC analysis, the NLR with a cutoff value of 5.86 showed the second best sensitivity (89.4%), specificity (87.8%), and AUC value (0.794) (as a diagnostic tool), and CRP showed the best sensitivity and AUC value (0.871) (Fig. 2).

**Table 6:**
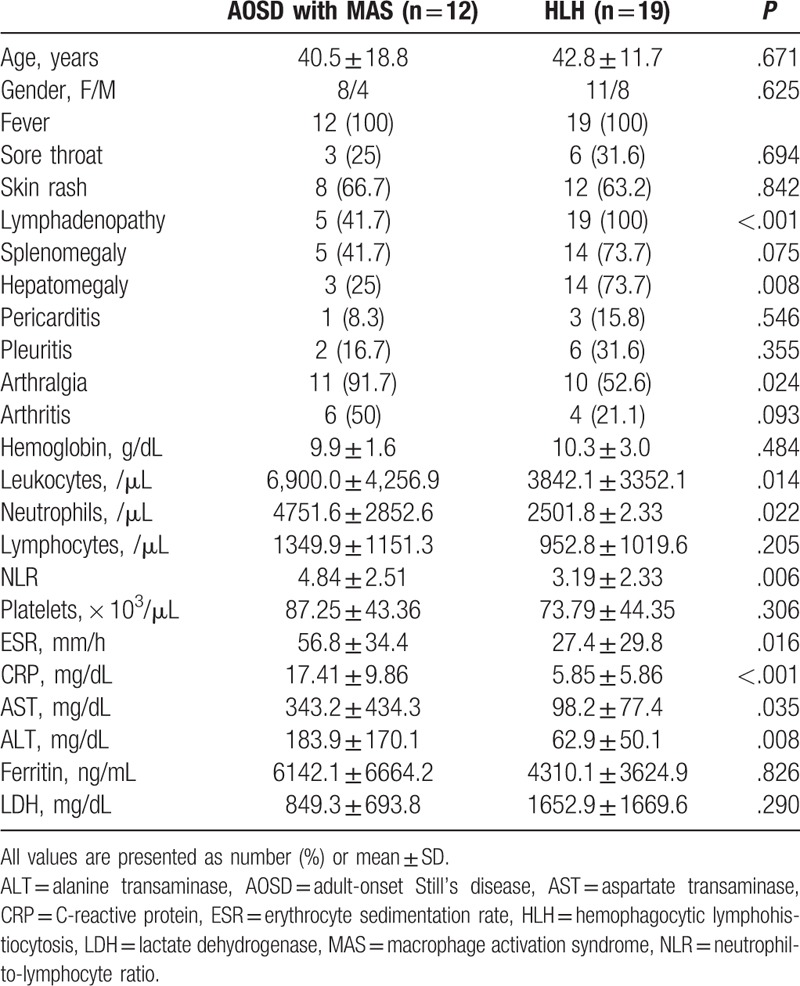
Clinical characteristics of patients for AOSD with MAS and HLH.

## 4 Discussion

This is the first study to evaluate the role of the NLR in AOSD. The results indicated that the NLR could be used to differentiate and diagnose AOSD. The NLR showed significantly higher sensitivity for diagnosis of AOSD than other inflammatory markers, such as ESR, CRP, and ferritin. Furthermore, the NLR was significantly higher in patients with MAS than in HLH patients, and the NLR with a cutoff value of 5.86 showed good sensitivity for discrimination between MAS and HLH.

AOSD is a rare systemic inflammatory disease and has several clinical manifestations, such as fever, sore throat, and skin rash.^[15]^ However, this disease does not have specific characteristic symptoms and laboratory results, and therefore diagnosis can only be made after exclusion of infection and cancer.^[16]^ There are several prognostic biomarkers of inflammation, including WBC, ESR, CRP, procalcitonin, IL-6, IL-8, and TNF-α, all of which have been reported as prognostic or disease activity markers in AOSD. However, simple inflammatory markers, including ESR and CRP, are nonspecific, and measurement of proinflammatory cytokines is usually inefficient and expensive. The NLR can be determined simply from neutrophil and lymphocyte counts and is an inexpensive biomarker. The NLR has been suggested as a useful biomarker for evaluation of the clinical status and clinical outcome in some diseases.^[17,18]^ Neutrophilia with lymphopenia is a response of the innate immune system to systemic inflammation.^[19]^ An elevated NLR is associated with the prognosis of systemic inflammatory diseases, especially infectious diseases.^[20–22]^ In this study, the clinical symptoms and blood test results of 164 patients with suspected AOSD were retrospectively collected, and 127 were shown to have AOSD. We compared the NLR between the AOSD patients and non-AOSD patients, and found that it was higher in AOSD patients than in non-AOSD patients. These results suggest that the NLR may be a useful additional tool for diagnosis of AOSD. However, we did not demonstrate an association between the prognosis of AOSD and an elevated NLR. The NLR was not related to the death rate of AOSD patients, but was related with relapse. Furthermore, we did not find any difference in NLR levels between different AOSD disease patterns. Although their clinical courses were different during follow-up, their initial manifestations and systemic scores were similar between the monophasic pattern and polycyclic or chronic articular patterns (data not shown). It could be affected to the initial NLR levels. Therefore, the NLR was not useful for predicting the prognosis of AOSD.

The NLR has also been reported as a disease activity marker in several inflammatory diseases.^[5,23–27]^ One study showed that the NLR was higher in RA patients compared to healthy controls.^[5]^ Furthermore, the NLR increased with worsening 28-joint Disease Activity Score (DAS28). In addition, the NLR and platelet-to-lymphocyte ratio were investigated in patients with RA, and these levels were found to be correlated with CRP and DAS28 in RA patients.^[27]^ The NLR was also studied in SLE patients, and was shown to be correlated with CRP, ESR, and SLE disease activity index scores.^[24]^ However, there have been no studies addressing the NLR in AOSD patients. This is the first report evaluating the NLRs of AOSD patients, including analysis and comparison of clinical features and pre-existing disease activity markers. The NLR was weakly correlated with the levels of some markers of disease activity, including the systemic score, ESR, and ferritin level. However, the NLR did not differ according to the presence or absence of clinical manifestations, such as skin rash or sore throat. These results suggest that the NLR is not a good marker for evaluation of disease activity in patients with AOSD.

In our study, some AOSD patients showed features of MAS, which is an overwhelming inflammatory reaction due to an uncontrolled immune response involving the expansion of macrophages and T lymphocytes.^[28]^ This reaction is activated continuously and results in massive hypersecretion of cytokines. MAS is pathophysiologically and clinically similar to HLH.^[29]^ HLH is not an independent disease, instead being a hyperinflammatory syndrome with high mortality that occurs in many underlying conditions. HLH is subdivided into primary and secondary HLH, and MAS is sometimes referred to as secondary HLH. HLH is diagnosed based on the diagnostic criteria developed by the International Histiocyte Society.^[30]^ In this study, we compared the clinical features and laboratory data of AOSD patients with MAS to those of patients with HLH. The NLR was shown to have a higher AUC in MAS compared to leukocyte and platelet counts. This result suggests that the NLR could be used as an additional tool to differentiate between HLH and MAS.

Our study had several limitations. First, we reviewed the clinical data retrospectively. Thus, some patient data may have been missing and were not available in a few patients. Second, there may have been selection bias because the data used were from a single center. Third, we did not compare the data of AOSD with those of healthy controls, and the sample size was relatively small for the HLH patients and AOSD patients with MAS. However, there have been many previous reports on the NLRs of healthy controls.^[5,24,26,27]^

In conclusion, although the NLR does not predict the disease activity or prognosis of AOSD, it can be used as an additional diagnostic tool and predictor of relapse, and for the differential diagnosis of HLH. Further studies in larger populations are required to confirm our results regarding the diagnostic role of the NLR in AOSD patients.
